# Adaptive algorithm for dependent infrastructure network restoration in an imperfect information sharing environment

**DOI:** 10.1371/journal.pone.0270407

**Published:** 2022-08-24

**Authors:** Alireza Rangrazjeddi, Andrés D. González, Kash Barker

**Affiliations:** School of Industrial and Systems Engineering, University of Oklahoma, Norman, Oklahoma, United States of America; University Campus Bio-Medico of Rome, ITALY

## Abstract

Critical infrastructure networks are vital for a functioning society and their failure can have widespread consequences. Decision-making for critical infrastructure resilience can suffer based on several characteristics exhibited by these networks, including (i) that there exist interdependencies with other networks, (ii) that several decision-makers represent potentially competing interests among the interdependent networks, and (iii) that information about other decision-makers’ actions are uncertain and potentially unknown. To address these concerns, we propose an adaptive algorithm using machine learning to integrate predictions about other decision-makers’ behavior into an interdependent network restoration planning problem considering an imperfect information sharing environment. We examined our algorithm against the optimal solution for various types, sizes, and dependencies of networks, resulting in insignificant differences. To assess the proposed algorithm’s efficiency, we compared its results with a proposed heuristic method that prioritizes, and schedules components restoration based on centrality-based importance measures. The proposed algorithm provides a solution sufficiently close to the optimal solution showing the algorithm performs well in situations where the information sharing environment is incomplete.

## Introduction

Infrastructure networks are complex systems related to flow, movement, or exchange of entities such as electric power, water and wastewater, data and information, and critical goods and services, among many others [1]. Such infrastructure networks are often interdependent, implying that their integration and connection means that the performance of one network can affect others [2–6]. These infrastructure networks are regularly becoming more connected due to synergic efficiencies obtained by creating highly interdependent systems [1, 7]. Therefore, maximizing the performance of an infrastructure network in isolation neglects the mutual benefits that each network gains from interdependency with other networks, as well as the basic reality of the connectedness of these networks [1, 7–9].

The proper operation of these interdependent infrastructure networks is crucial for a functioning society, particularly from the perspective of economic productivity, public health, and national security, among others [8, 10, 11]. Therefore, the restoration of disrupted infrastructure networks has been an important area of study. The traditional approach in solving such problems is to follow a top-down, centralized process, which is more suitable for government strategy developers dictating a solution [12]. As a result, many models and algorithms have been developed to determine restoration schedules for disrupted networks from a centralized perspective, where a complex system is controlled by an overarching decision-maker [13–20]. However, in many real cases, the restoration of infrastructure networks is scheduled in decentralized manner, as different networks are controlled by different entities (e.g., utility companies), potentially autonomous and potentially with conflicting interests [9, 21–24]. As such, game theory can be effective in describing the effect of the selection of different strategies by one decision-maker on those of another.

Further, oftentimes deterministic assumptions are made about various aspects of the restoration scheduling problem. Such assumptions may not be realistic as in many real decision environments, there exists uncertainty due to a lack of information. This is especially true in the time of disasters in which there is a lack of complete information with which to evaluate the magnitude of the events and resources available for decision-makers across the system of networks [9, 25–28]. Without proper information, decision-makers cannot easily anticipate the preference of other decision-makers and their approach to the restoration problem. Generally, information regarding the functional status of various components in a disrupted system of networks is dynamically accumulated over time while the network is gradually recovered [29].

This paper proposes an iterative algorithm using ideas from (i) dictatorial game theory [30] to address the decentralized environment of restoring disrupted interdependent infrastructure networks and (ii) machine learning techniques to predict the restoration actions of other decision-makers under an imperfect information sharing environment. That is, the objective of the proposed algorithm for the follower player is to adapt to the network restoration movements of the leader player in the future when the leader is not willing to share or disclose their restoration schedule for interdependent components. The algorithm employs a modified version of the time-dependent interdependent network design problem (td-INDP) [31] to calculate the best response given the available prediction of the other decision-maker actions. The key attributes of the proposed algorithm are: (i) each network decision-maker (player) is considered to be self-interested, responsible only to minimize the unmet demand of their network, (ii) only one-way interdependency among networks is considered, so that the proposer player dictates their strategy selection to the responder player (representing a dictatorial game), and (iii) the responder player is not aware of the decisions that have been taken by the proposer player and needs to predict the proposer’s actions using some features related to the network topology utilizing a machine learning model. Studies in this field have made contributions by considering various assumptions, such as showing cooperative nature among decision-makers where each decision-maker shares its plan with others until they converge into agreement [32], or by being informed gradually from other decision-makers actions without having any prediction of others action [9]. Although both studies illustrate the different perspectives of the problem, the utility decision-making often lies somewhere between contrasting assumptions of full information availability and no information sharing. Generally, neither players in the same decision-making environment have full access to the information of the status of the entire system, nor are they completely uninformed. In the proposed algorithm, we have considered a more realistic approach for predicting the moves of other decision-makers and then incorporating the prediction in the decision-making process as definite information gradually becomes available. Although machine learning is used in various studies, its application in the restoration scheduling of the disrupted interdependent infrastructure networks in a decentralized environment is unique in this paper.

The key contributions of the proposed algorithm are: (i) improving the ability of the responder player to be effectively and dynamically adaptive to the proposer player selected strategies, (ii) combining definite information and the predicted restoration schedule of the other decision-makers using a machine learning approach, and (iii) determining the best response strategy for the responder considering the accuracy of the prediction for the proposer’s action. In simple terms, the best strategy response can be achieved for the responder if the proposer selected strategy can be predicted accurately. Therefore, the higher the accuracy of the prediction, the less costly strategy can be selected for the responder player. The remainder of this paper is structured as follows. The Literature Review section offers an overview of decentralized decision-making approaches. The methodology to develop the proposed algorithm is offered in the next section. Then the performance and capability of the algorithm is explored. Finally, concluding remarks wrap up the paper.

### Literature review

Decentralized management has recently been applied to model the control of today’s complex systems and infrastructure networks [9, 22]. Researchers have studied the use of decentralized optimization in various practical domains such as path exploration systems for autonomous vehicles [33–35], air traffic management [36], resource allocation in networks [37], and sensor network management [38], among others. Generally, a decentralized optimization approach deals with problems of multiple objectives that each maximize the interests of a specific decision-maker [22, 26, 39, 40]. This decentralization can be the result of either having variation among the goals of different decision-makers in a non-cooperating manner or due to the lack of clear information among them regarding their preference in a cooperative/semi-cooperative environment.

Furthermore, researchers have extensively employed deterministic and stochastic decentralized convex optimization to address decentralized decision problems. Tsitsiklis [41] and Tsitsiklis et al. [42] investigated how like-minded decisions can be made in a decentralized environment utilizing iterative optimization algorithms given delays in communication among decision-makers. Some researchers have focused on optimizing the sum of local functions, performing an averaging process for each decision-maker while descending stepwise along the local sub-gradient direction considering their local constraints [43–48]. The general drawbacks with this approach are slow convergence and lack of accuracy [49], therefore the alternating direction method of multipliers (ADMM) was used by Boyd et al. [50] to achieve linear convergence in exchange for higher computation time. Consequently, for improved computation time, linearized ADMM algorithms have been utilized by Ling et al. [49]. These methods emphasize the cooperative nature of the decision-makers to optimize a global objective function (i) that each understands only partially (ii) while their communication is also not perfect and occurs in time intervals. Note that the cooperative nature in a decision-making environment exists when only one objective function is optimized while each decision-maker in the system can control certain variables of the main problem. Therefore, considering the large size of the private market where these companies do their best to maximize their profit regardless of the others (non-cooperative nature), cooperative behavior becomes less practical in a free-market environment.

A Markov decision process (MDP) is another approach that has been utilized to investigate the problem of decentralized decision-making [39, 51–53]. Using the concept of MDP, Åström [54] introduced the partially observable MDP (POMDP) for centralized sequential decision-making, which is appropriate in the imperfect information environment. In this regard, the decentralized partially observable MDP (DEC-POMDP) is the generalized version of the POMDP that accounts for problems with two or more decision-makers available in the system cooperating in an imperfect information environment [22]. Similar to decentralized convex optimization, these players in the system cooperate to optimize the joint reward function while each has its different observation function [39]. Unfortunately, studies showed the significant difficulty of solving the DEC-POMDP problem for multiple decision-makers compared to the single agent POMDP problem. It turned out that the DEC-POMDP problem finite-horizon is exponential time complete (NEXP-complete) when only two decision-makers are available in the system [55]. Therefore, considering the various forms of the MDP approach, the methodology is substantially time-intensive for problems with more than one decision-maker in the system.

Game theory represents a different approach that focuses on the behavior of different decision-makers in a system with a cooperative/non-cooperative nature that has gained popularity in solving problems with network characteristics [9, 11, 23, 26, 56]. Tosselli et al. [26] developed a repeated-negotiation game approach considering a conceptual enterprise model. The approach deals with two types of multi-layer project and resource decision-makers that are responsible for the scheduling of tasks and resources, respectively. Decision-makers maximize their objectives related to the project deliverables and resource utilization metrics having a client-server relationship. Their algorithm could be considerably time consuming as it uses repeated negotiations to converge to a Nash equilibrium or a close Nash equilibrium solution. Also, instead of predicting the other players’ move, the algorithm considers a certain behavioral assumption which is altered according to a predefined probability in each iteration, and such an assumption could affect the practicality of the algorithm. Huang and Zhu [56] proposed an iterative algorithm based on the dynamic game framework to investigate the interactions between a stealthy attacker and a proactive defender related to the security of a cyber-physical system. Although this study does not provide a methodology for scheduling the recovery of a disrupted network, it presents a Bayesian game-theoretic algorithm for detecting the malevolent attacker of the cyber-physical system. The iterative algorithm assumes that it can observe all interactions the users/attackers can make in each iteration related to the system. Therefore, the defender of the system has access to the real-time information of all other users/attackers, which may not be the case in practice. Sharkey et al. [32] considered the cooperative nature among decision-makers, who find a resolution for their payoff function considering optimistic/pessimistic assumptions and share their plan for recovery with other decision-makers to update their assumptions and find new resolutions for their payoff function. However, the proposed framework assumes an unlimited number of negotiations between players related to their recovery plans, which is time consuming and may not to be converged in some cases since there is no upper bound for the number of negotiations [9]. Although several centralized approaches have been suggested by the researchers to address the problem of interdependent infrastructure network recovery, only a few works have used game-theoretic models to address the decentralized nature of decision-making in such networks. Smith et al. [9] developed an ad hoc sequential game-theoretic model to address the restoration problem of interdependent networks in a decentralized environment. They utilized the reduced version of the interdependent network design problem (INDP) model developed by González et al. [19] as the payoff function for the decision-makers of the system. The computational time of their algorithm could be sensitive to the availability of resources and the number of disrupted components related to each decision-maker in the system. This is because the algorithm must consider all available strategies in each period and keep track of the total cost through the decision path in the whole problem time horizon. Subsequently, the algorithm calculates the Nash equilibrium using backward induction, which can be time-consuming as all possible outcomes of strategy combinations are enumerated. Also, the assumption that decision-makers act in turn weakens the algorithm’s practicality. Furthermore, considering only definite information about the actions of the other decision-makers is the other drawback of this research. Note that communication among decision-makers and the quality of the exchanged information is important in interdependent infrastructure network recovery problems [9, 22, 32, 57]. Furthermore, Smith et al. [9] assumed decision-makers are unaware of the status of nodes on which they depend in the other networks controlled by other decision-makers, and they will be updated gradually when they are recovered. Therefore, although they do not have complete information about the other decision-makers, the information that is available to the decision-makers is considered definite. However, our proposed algorithm combines definite information and the best guesses of decision-makers in their decision-making process to achieve a better result. In addition, the proposed algorithm is an efficient framework for predicting future decision-maker behavior and devising an adaptive plan concerning the recovery of the disrupted interdependent infrastructure network. Therefore, with the aid of this method, an acceptable solution can be achieved efficiently following a disruptive event. We present the details of the proposed algorithm in the following section.

## Interdependent network adaptive recovery model

It is important to determine the method for evaluating the benefits that each decision-makers gain relative to their objectives. Regardless of the number of decision-makers in the system, researchers have approached the network restoration problem from various angles. The main objectives generally drive the restoration of infrastructure networks is to restore the network components as quickly as possible so that user demand from the network is reinstated. In this regard, some have designed their objective function to impose a cost for every period in which user demand is not satisfied [15, 16, 19, 20, 58–61]. The other angles of this problem that have been generally illustrated in the constraints of these models are (i) network flow cost, (ii) interdependency, (iii) crew constraints, and (iv) resource and budget constraints, among others. In these models, to be efficient in any of these perspectives, some costs have been associated with them and included in the objective function [14]. They have considered different perspectives that exist in practical situations to mirror essential characteristics in real network disruption scenarios. The interdependent network adaptive recovery (INAR) algorithm proposed in this paper can adapt with the variety of these models predicting the proposer’s network actions and implementing them in the responder network’s optimization model to devise the best response action. In this research, we considered a modified, shortened version of the INDP model developed by González et al. [19] to calculate the best response given different predictions.

The algorithm is based on the continuous prediction of opponent player behavior, adaptive strategy optimization, and updating assumptions with new information. We assumed that each decision-maker has only full information about the network that they are responsible for, yet their awareness of the other networks is limited to that network’s general topology and the importance of its components related to its supply-demand capabilities. Aggregating our assumption using opponent network features and considering the dependencies between the networks, we used a machine learning approach to predict the opponent player recovery schedule for subsequent periods. Therefore, the recovery process of the opponent’s network can be approximated. As such, the responder can anticipate when to expect the dependency relationship to be resolved by the opponent player. Having a good guess for the opponent strategic recovery schedule enables the decision-makers to incorporate this indefinite information into their optimization models as parameters and constraint. Therefore, the higher the responder’s prediction, the better the strategic reaction, and the closer it would represent the best and ideal response. We assumed that the responder could be informed of an interdependent component recovery as soon as the proposer recovers it, thus the deviation of the predicted recovery schedule from the actual can be realized as time passes. As the recovery process of disrupted infrastructure networks is developed over time, the predictions made at the beginning of the planning time horizon could be significantly biased from the opponent’s actual strategic plan. Therefore, as the recovery process progress and information gradually aggregate, we need to renew the prediction to minimize the deviation between the predicted and actual plans.

**Fig 1:**
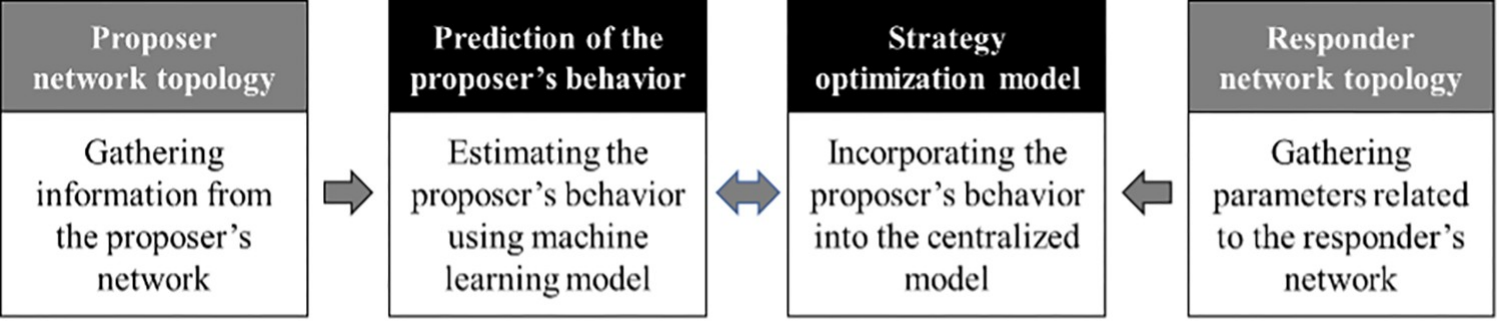
General overview of the proposed algorithm.

Fig 1 provides a general overview of the proposed algorithm. Note that the algorithm breaks the problem into two parts of prediction and strategy optimization. Therefore, the first level predictions are inserted into a centralized recovery model as parameters and constraints to find the best reaction response.

In the following three sub-sections, we describe (i) the prediction model characteristics, including the data preprocessing and the learning process of the prediction model, (ii) the optimization model responsible for finding the best response considering the available information, and (iii) the algorithm for updating the strategy, including the continuous strategy correction. Table 1 summarizes several acronyms used in our discussion, as well as the notation for sets, parameters, variables, and terms involved in Algorithms 1 and 2.

**Table 1:** Summary of acronyms, sets, parameters, variables.

Acronyms	Meaning
INDP	Interdependent network design problem
td-INDP	time-dependent interdependent network design problem
mtd-INDP	Modified time-dependent interdependent network design problem
INAR	The interdependent network adaptive recovery
PCA	Principal component analyses
Index	Meaning
	Index for the components in the networks
*t*(*i*, *j*)∈*N*	Index for the time in the recovery time horizon
*l*	Index for the type of the commodity
Sets	Meaning
*T*	Set of time periods in recovery time horizon
*T*′	Set of time periods where a disrupted interdependent component is predicted not be recovered in the proposer’s network
$T_{a}^{*}$	Set of actual earliest recovery times for the interdependent components
$T_{p}^{\prime }$	Set of predicted earliest recovery times for the interdependent components
*N*	Set of all components in the network
*N*′	Set of disrupted components in the network
$\hat{N}$	Set of disrupted interdependent components in responder’s network
$N_{I}^{\prime }$	Set of all interdependent components in the network
*A*	Set of links in the network
*L*	Set of commodities
*R*	Set of resources
Parameters	Meaning
*c*_*ijlt*_	Unit flow cost of commodity *l* through link (*i*, *j*) at time *t*
*e*_*it*_	Recovery cost of component *i* at time *t*
$M_{ilt}^{+}$	Unit cost of component *i* excessive supply at time *t* for commodity *l*
$M_{ilt}^{-}$	Unit cost of component *i* unmet demand at time *t* for commodity *l*
*b*_*ilt*_	Supply/demand of component *i* at time *t* for commodity *l*
*u*_*ijt*_	Capacity of link (*i*, *j*) at time *t*
*h*_*irkt*_	Required resource for recovery of component *i* at time *t*, from resource *r*
*v*_*rt*_	Available resource *r* at time *t*
Variables	Meaning
$\delta_{ilt}^{+}$	Excessive supply of component *i* at time *t* for commodity *l*
$\delta_{ilt}^{-}$	Unmet demand of component *i* at time *t* for commodity *l*
*f*_*it*_	Functionality of component *i* at time *t*
${\tilde{f}}_{it}$	Extent of recovery of component *i* at time *t*
*x*_*ijlt*_	Flow on link (*i*, *j*) in network *k* and time *t*
Algorithm terms	Meaning
$i_{t}^{*}$	Index of the component that is recovered in time *t*
*t*_*i*_	Recovery time of the disrupted component *i* in the recovery time horizon
*t*_*p*_	Current time of the algorithm
*cn*_*i*_	Centrality metric for component *i*
*rc*_*it*_	Rescaled centrality metrics for disrupted component *i* relative to the other disrupted components in time *t*
$S_{i}^{p}$	Minimum element in set $T_{p}^{\prime }$
$S_{i}^{a}$	Minimum element in set $T_{a}^{*}$
*cls*_*it*_	Actual recovery status of component *i* in time *t*

### Prediction model

Adopting the Markov decision process concept that the best policy in a specific state is selected, the proposed algorithm predicts the component from the opponent network that is recovered in the next period. That is, the predictive model should classify whether the proposer recovers any specific disrupted node in the next period. The model checks every remaining disrupted component in each period and see if it is the next candidate for recovery. Hence, we employed the random forest ensemble model to cluster each component into two classes in each period: recovered and not recovered.

Beginning with Wollmer [62], who proposed an algorithm to find sensitive components of a network by removing a number of them to maximize the flow reduction in a network, numerous component importance measures have been developed to identify critical components in a network. To approximate each component’s importance in the network, we used several network centrality measures as the predictors of our random forest model. We are motivated by the fact that, although decision-makers are not aware of the opponent player reward function and constraints, generally, they can assume that the components with higher importance are recovered earlier relative to others in the network. So, the service flow through the network can be replenished faster and unmet demand costs could be reduced. Additionally, we determined the actual target classes using a *modified* version of td-INDP model, referred to as the mtd-INDP. In fact, when we find the solution of mtd-INDP model for the proposer’s network recovery, then we can easily realize the class to which each node belongs at each iteration.

The following stages were used to prepare the data, preprocessing, and learning the random forest model: (i) perform data preparation considering various disruption scenarios, (ii) apply principal component analyses (PCA), and (iii) learn the random forest ensemble model using the prepared data and calculate the model accuracy. In the data preparation stage, we balanced the data using the under-sampling method [63] to reduce the size of the abundant class to improve the random forest model and more effectively distinguish the minority class. PCA was used to make the clustering process easier for the random forest model [64]. We used the random forest model for this study as it can provide accurate and efficient results for both prediction and clustering problems considering a variety of applications [65, 66].

While the algorithm could be implemented with any number or selection of importance measures, we selected six network centralities due to their ubiquity in the literature: (i) degree, (ii) betweenness, (iii) closeness, (iv) Katz, (v) page-rank, and (vi) load. Naturally, different topological measures might be more useful than others depending on how importance is being defined [67]. However, note that it has been shown that in many instances topological measures are sufficient surrogates for different (and more difficult to estimate) flow-based measures [68]. Each of these centrality measures has been defined to represent the network components importance from various perspectives. Degree centrality represents the connection of nodes to other nodes in the network. Betweenness centrality refers to the number of shortest paths in the networks that pass through each component. Closeness centrality considers the sum of the shortest path length between one node and all other nodes in the network. Katz centrality measures the number of immediate neighbors of a node and the other nodes that connect to the node through its immediate neighbors to compute the relative influence of that node in the network. Page-rank centrality is an extension of the Katz centrality, considering the advantage a node can receive connecting the network’s critical nodes. The load centrality for a node is calculated considering the number of all shortest paths passing through the node, assuming each node sends a hypothetical unit of some materials to its neighbors.

Furthermore, in each period of recovery time horizon, these metrics are rescaled considering only the disrupted components and serve as the input variables for the classification model. Additionally, we considered the nominal demand/supply capacity of each component as another predictor, as such capacities may also provide insight on the importance of the component.

Algorithm 1. Data processing procedure.

**1. Input:** proposer’s network relationships and parameters

2. Calculate the component actual recovery sequence using the mtd-INDP model (i.e., component actual recovery time in the set $\left\{t_{i}^{*}:t\in T,\;i\in N^{\prime }\right\}$)

3. Calculate the component centrality metrics (i.e., component centrality in the set {*cn*_*i*_:*i*∈*N*})

4. **for** *t* in *T* **do**

5.    Rescale the centrality metrics (*rc*_*it*_) for all *i*∈*N*′, for time *t*

6.        **if** $i=i_{t}^{*}$ **then**

7.            Set actual component class (*cls*_*it*_) = 1

8.            Remove *i* from *N*′

**9.        Else**

10.                Set actual component class (*cls*_*it*_) = 0

11.    **Output:** Rescaled centrality metrics corresponded to the actual recovery status of the component for every component in each period {(*rc*_*it*_, *cls*_*it*_):*i*∈*N*′,*t*∈*T*}

The data processing algorithm (Algorithm 1) shows the data preparing process schematically, where *T*, *N*, and *N*′ are the set of periods in the recovery time horizon, the set of all components in network, and the set of all disrupted components in the network, respectively (discussed in Table 2 in the context of the restoration optimization problem). The notation *cn*_*i*_ and *rc*_*it*_ represent the array of different centrality metrics calculated for each component and the array of rescaled component centralities considering only disrupted components, respectively. Note that whenever each component is restored, it is required to be removed from the set of disrupted components in each period. Therefore, the remaining component centrality metrics should again be rescaled to keep the summation domain of each centrality metric between [0, 1]. This way, we also keep updating the priority of each remaining disrupted component related to each centrality metric. Index *i* is associated with the components in the network, while $i_{t}^{*}$ in Algorithm 1 is the component of the network that is candidate for recovery in specific period of *t*. The cluster to which a component belongs in a specific period is referred to as *cls*_*it*_. Note that *cls*_*it*_ shows whether or not component *i* is recovered in time *t*.

**Table 2:** Illustration of variables and an example of prepared data.

Node	Betweenness	Page-rank	Katz	Closeness	Degree	Load	PC1	PC2	PC3	Cluster (*cls*
78	0.012	0.013	0.015	0.021	0.012	0.003	418	0.031	0.02	1
38	0.012	0.013	0.018	0.023	0.012	0.002	0	0.061	-0.009	0
30	0.025	0.025	0.021	0.025	0.024	0.026	-112	0.081	0.013	0
44	0.019	0.025	0.018	0.023	0.024	0.015	-401	0.1	-0.021	1
23	0.025	0.035	0.028	0.027	0.036	0.025	-5	0.107	0.01	1
10	0.102	0.082	0.059	0.034	0.09	0.157	-522	0.219	0.137	0
102	0.014	0.014	0.019	0.025	0.013	0.004	492	0.092	-0.019	0
16	0.015	0.014	0.022	0.028	0.013	0.004	0	0.104	-0.035	1
53	0.013	0.015	0.016	0.023	0.013	0.002	-17	0.101	-0.039	0

After preparing data using the algorithm, PCA is performed on the data to find the predictors that capture the greatest variance. These principal components are added to the predictors for our random forest classification model. As we use a classification model categorizing the node’s status individually, the random forest model can mistakenly categorize several components into the recovered class in each period. To address this concern, in case of having several candidate components to recover in a specific time, we will use the probability produced by the random forest model for each of those categorized components. We select the component with higher probability as the recovered component in each period, as we consider having the higher chance of correct classification.

### Strategy optimization

After predicting opponent recovery behavior, these predictions are integrated into the strategy optimization. In this study, we were inspired by the td-INDP model developed by González et al. [31] which focused on the recovery of disrupted components in interdependent networks in a centralized manner and with full information about all networks. We have modified the td-INDP model to suit the proposed algorithm in this study. As the proposed algorithm assumes multiple players in the system in an imperfect information sharing environment, we need to address the interdependency perspective differently. Thus, the equations satisfying the interdependency relationships of the networks are no longer required for the proposed algorithm’s assumptions (in the INAR algorithm, the interdependencies among networks are addressed by adding constraints representing the responder’s prediction for when interdependent disrupted component is recovered in the proposer’s network). The td-INDP also accounts for geographical constraints, focusing on minimizing the land preparation fixed cost, which we found deviant from the general practices in network recovery problems. Therefore, pruning the td-INDP model, we focused on the constraints responsible for minimizing the cost of unmet demand, excess supply, and network flow, considering a single-layered network environment.

The objective function of the mtd-INDP model in Eq (1) consisted of three different terms representing the costs for (i) commodity flows represented by *x*_*ijlt*_, (ii) component recovery represented by ${\tilde{f}}_{it}$, and (iii) unmet demand and excessive supply represented by $\delta_{ilt}^{-}$ and $\delta_{ilt}^{+}$, respectively. Note that the ${\tilde{f}}_{it}$ is a binary variable showing if the disrupted component *i* is recovered in time *t*.


$$min\;Z=\sum_{t\in T}\left[\sum_{l\in L}\sum_{(i,j)\in A}{c_{ijlt}x}_{ijlt}+\sum_{i\in \overset{´}{N}}{e_{it}\tilde{f}}_{it}+\sum_{l\in L}\sum_{(i,j)\in A}{(M}_{ilt}^{+}\delta_{ilt}^{+}+M_{ilt}^{-}\delta_{ilt}^{-})\right]$$ (1)

Also, six groups of constraints have been considered for the model that govern the following: (i) the balance of goods/services flow through the network in Eq (2), (ii) the activation mechanism for disrupted components in Eq (3), (iii) the link capacities in Eqs (4) and (5), (iv) the availability of resources in Eq (6), and (v) enforcing disruption to the network for the first period in Eq (7). The balance constraint in Eq (2) governs that the commodity output and input outcomes be equal to the amount of consumption, unmet demand, and excessive supply. Constraint (3) is responsible for keeping the disrupted components non-functional until recovered. Constraints (4) and (5) prevent flow in link (*i*, *j*) if either node *i* or node *j* are not functional. The nature of the decision variables is described by Eqs (8)–(10).


$$\sum_{j:(i,j)\in A}x_{ijlt}-\sum_{i:(i,j)\in A}x_{ijlt}=b_{ilt}-\delta_{ilt}^{+}+\delta_{ilt}^{-}\;\forall i\in N,\forall l\in L,\forall t\in T$$ (2)

$$f_{it}\leq \sum_{t\in T}{\tilde{f}}_{it}\;\forall i\in N\prime ,\forall t\in T$$ (3)

$$\sum_{l\in L}x_{ijlt}\leq u_{ijt}f_{it}\;\forall (i,j)\in A,\forall t\in T$$ (4)

$$\sum_{l\in L}x_{ijlt}\leq u_{ijt}f_{jt}\;\forall (i,j)\in A,\forall t\in T$$ (5)

$$\sum_{i\in \overset{´}{N}}h_{irt}{\tilde{f}}_{it}\leq v_{rt}\;\forall r\in R,\forall t\in T$$ (6)

$$f_{i0}=0\;\forall i\in N\prime$$ (7)

$$x_{ijlt}\geq 0\;\forall (i,j)\in A,\forall l\in L,\forall t\in T$$ (8)

$$\delta_{ilt}^{+},\;\delta_{ilt}^{-},\geq 0\;\forall i\in N,\forall l\in L,\forall t\in T$$ (9)

$$f_{it}{,\;\tilde{f}}_{it}\in \{0,1\}\;\forall i\in N,\forall t\in T$$ (10)

To include the restoration predictions in the model, two approaches can be considered. In the first approach, it is assumed that the responder does not attempt to recover the interdependent disrupted components until its corresponding node in the proposer’s network is fixed. This is governed by inserting Eq (11) into the optimization model. Alternatively, in the second approach, the model recovers the disrupted node but it cannot be activated until its predecessor in the proposer’s network are restored. To adopt this approach, Eq (12) is instead inserted into the model.


$$\sum_{t\in \overset{´}{T}}{\tilde{f}}_{it}=0\;\forall i\in N\prime$$ (11)

$$\sum_{t\in \overset{´}{T}}f_{it}=0\;\forall i\in N\prime$$ (12)

### Best response and strategy update

Using the random forest model predictions of the proposer ’s behavior with recovered and not recovered classes for each node, we need to find the best response to the proposer ’s behavior considering the mtd-INDP model. The best recovery schedule of the responder’s disrupted nodes is found. Although we can make predictions at the primary stage of the recovery time horizon, as time advances, decision-makers acquire more information about the actions made by the opponent player. These pieces of information generally describe the interdependent components that have been recovered. Thus, the responder can simply realize the deviation between their prediction and actual actions made by the proposer. As this information updates gradually, the prediction can be renewed and subsequently the responder can more effectively adapt to the proposer’s decisions whenever they receive new information.

Gathering such new information could differ by the decision-making situation, the level of cooperation among decision-makers, and the cost of information. In this research, it is assumed that the responder player predicts the proposer’s behavior at the beginning of the time horizon and plans the recovery of the network on that prediction. Then, as the responder pursues the actual recovery plan over time, they will be informed whenever the proposer recovers an interdependent component necessary for functionality of a node in their network. Based on this assumption, three different situations may happen. The first is that the prior prediction is identical to the proposer’s actual recovery plan. The second is that an interdependent component is recovered sooner than expected. The third situation is that an interdependent component is not recovered by the expected time. Considering these situations, if the prediction is not identical with the actual plan, the proposer needs to consider the new condition, renew its prediction, and develop a new recovery plan for the rest of the remaining periods. The strategy updating algorithm (Algorithm 2) shows the strategy update procedure in detail.

Algorithm 2. Strategy updating algorithm.

1. **Input:** proposer’s and responder’s network relationships and parameters

2. Setting the actual earliest recovery time set ${(T}_{a}^{*})$ for the interdependent components using mtd-INDP model on proposer’s network: $T_{a}^{*}=\{t_{i}^{*}|t\in T,i\in N_{I}^{\prime }\}$

3. Setting the predicted earliest recovery time $\left(T_{p}^{\prime }\right)$ for interdependent components: $T_{p}^{\prime }=\{t_{i}|t\in T,i\in N_{I}^{\prime }\}$

4. Run mtd-INDP model to find the best recovery schedule set for the responder’s network considering the prediction set $T_{p}^{\prime }$

5. **While** $N_{I}^{\prime }!=\emptyset$ **do:**

6.    Select the minimum *t*_*i*_ from the set $T_{p}^{\prime }:S_{i}^{p}=min(T_{p}^{\prime })$

7.    Select the minimum $t_{i}^{*}$ from the set $T_{a}^{*}:S_{i}^{a}=\min\left(T_{a}^{*}\right)$

8.    Set the algorithm time (*t*_*p*_): $t_{p}=\{\min\left(S_{i}^{p},S_{j}^{a}\right)|i,j\in N_{I}^{\prime }\}$

9.    **If** $S_{i}^{p}<S_{j}^{a}$

10.        **If** component *i* status is recovered

11.            Remove *i* from $N_{I}^{\prime }:i\backslash N_{I}^{\prime }$

12.            Remove $t_{i}^{*}$ from $T_{a}^{*}$: $t_{i}^{*}\backslash T_{a}^{*}$

13.        Renew the interdependent component recovery time prediction set $T_{p}^{\prime }$

14.        Run mtd-INDP model to find the best recovery schedule set for the

    responder’s network considering the prediction set $T_{p}^{\prime }$

15.    **If** $S_{i}^{p}>S_{j}^{a}$

16.        Remove *i* from $N_{I}^{\prime }$: $i\backslash N_{I}^{\prime }$

17.        Remove $t_{i}^{*}$ from $T_{a}^{*}$: $t_{i}^{*}\backslash T_{a}^{*}$

18.        Renew the interdependent component recovery time prediction set $T_{p}^{\prime }$

19.        Run mtd-INDP model to find the best recovery schedule set for the

        responder’s network considering the prediction set $T_{p}^{\prime }$

**20.    Output:** Disrupted components recovery schedule set for the responder’s network

Note that both proposer and responder are working concurrently to recover the disrupted nodes in their network. If the responder realizes a fault in the prediction in any period, it has the chance to revise their plan for only the remaining disrupted nodes that have not recovered yet. Therefore, although the INAR algorithm’s solution is sub-optimal, renewing the predictions whenever we realize a conflict between the actual and prediction makes the solution stay close to the optimal as best as possible. Also, the algorithm terminated whenever no disrupted nodes remained in the disrupted interdependent component list ($N_{I}^{\prime }$).

Note that the running time of the prediction model is insignificant, as the prediction for the proposer’s independent components can be acquired almost instantly. Also, as the mtd-INDP model is the simplified version of the td-INDP model and the interdependency relationships between the networks are defined using simple constraints, the time required to solve the model is insignificant. Therefore, running algorithm 2 should not be time consuming at all.

## Results and discussion

**Fig 2:**
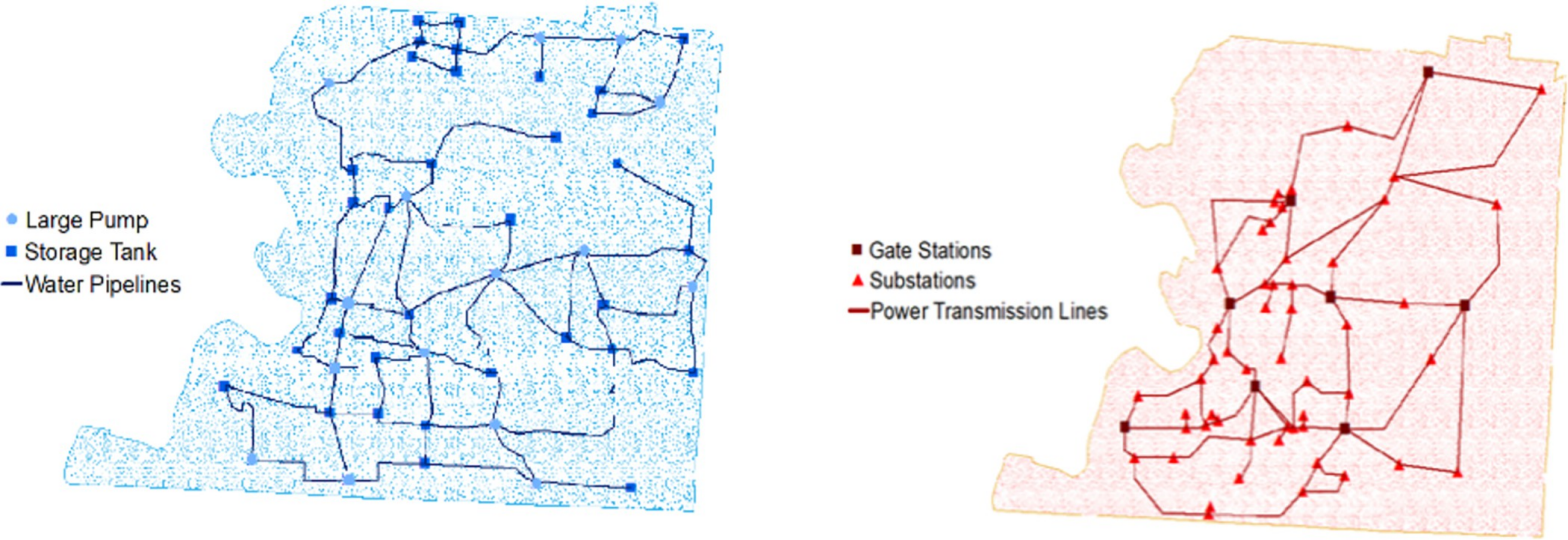
Shelby County, TN network topology for the (a) water and (b) power networks (adapted from González et al., (2016)).

To adequately explore the capability of the INAR algorithm, we applied this framework to four different case studies, one related to a realistic testbed and three randomly generated instances with different topological properties. The first case study is associated with the interdependent system in Shelby County, TN, USA [19, 69, 70]. To test the performance of our algorithm, as we have two decision-makers (each responsible for the restoration of a utility network) we focus our Shelby County case study on its water and power networks. The water and power networks have 49 and 60 nodes and 71 and 76 links, respectively. Fig 2 illustrate a general overview of Shelby County, TN water (a) and power (b) network.

The three remaining case studies correspond to: (i) Barabási-Albert network [71], (ii) Erdös-Renyi network [72] and (iii) Newman-Watts-Strogatz [73]. These three types were selected due to their popularity among the random network generation algorithms and for their varied structural properties. The Barabási-Albert algorithm generates a scale-free network where degree distribution of components follows the power law [71]. The Erdös-Renyi algorithm, one of the simplest random generation methods, produces networks with a binomial degree distribution [74]. The Newman-Watts-Strogatz network generation algorithm, derived from the Watts-Strogatz algorithm, produces networks with small-world properties [75]. The generated networks consisted of 120 nodes for each proposer and responder network, while the number of links varied based on the type of network generation algorithm. To keep the combinations of proposer and responder networks manageable, the pairs of interdependent proposer/responder networks are both generated by the same algorithm (e.g., a Barabási-Albert network paired with another Barabási-Albert network). Also, the interdependent nodes from both networks are selected and paired randomly. Note that the Barabási-Albert and Newman-Watts-Strogatz algorithms produce a connected network while the Erdös-Renyi network can create a disconnected algorithm based on the selected probability for link creation. To address this issue, the probability of link creation is increased incrementally so that only one connected component is produced. To generate these random networks, we use the networkx package in Python [76]. The number of links generated using the Barabási-Albert, Erdös-Renyi, and Newman-Watts-Strogatz algorithms are 273, 305, and 173 bidirectional links, respectively. The Shelby County networks, along with the three algorithm-generated networks, make up the four network types studied subsequently.

For each of the network types, 600 random disruption scenarios were generated, each with 40 disrupted components. Using the data processing algorithm (Algorithm 1), the data required for learning the random forest model were prepared for all four network types. Table 2 shows an example of prepared data for learning the random forest model. Columns 2 through 7 represent the rescaled value of the node’s centrality measures. Columns 8 through 10 are the principal components that have been calculated considering the centrality measures along with the amount of demand or supply for the node. Note that we have only used three principal components as predictors for learning the random forest model.

We have considered the ideal situation, where the responder is able to observe the proposer’s recovery plan beforehand as the comparison baseline. Furthermore, we evaluate the difference between (i) the solutions from the INAR algorithm with (ii) the ideal condition. As many works related to the network disruption have analyzed the network vulnerability by removing the network components based on centrality metrics [77–82], we compared the INAR algorithm solution to a restoration scheduling heuristic based on the ranking of the disrupted components according to individual centrality measures, as one might expect a decision-maker to choose important nodes to recover first.

Subsequently, we discuss the performance and validity of different parts of the INAR algorithm and describe its behavior. First, the performance of the random forest classification model for individual components in each period is described with different accuracy matrices. Then, we studied the INAR algorithm performance in predicting the recovery of the entire chain of the disrupted interdependent components in the proposer’s network. Finally, we investigate how a better prediction can affect the network recovery cost.

### Random forest classification performance

After completing the data preparation, we used the under-sampling method to balance the data by randomly deleting data from the majority class to the extent that the no information rate is less than roughly 52%, suggesting that the data do not suffer from imbalance issues that might lead to bias in the application of the machine learning techniques. Afterward, we use 20% of the data as the test data to validate the random forest model and calculate accuracy metrics. Table 3 shows several evaluation metrics illustrating the performance of the random forest model in classifying the restoration of each disrupted node in every period. The model accuracy for the four types of networks used ranges around 82–83% with low variability. As the no information rate for all different networks is around 51%, the random forest model operates at least 30% better than the random classification of disrupted component recovery status. As the training data were balanced with the proportion of each class being roughly 50%, the log-loss no information rate is roughly 0.69. The random forest model results in an improved log-loss accuracy ranging from 0.38 to 0.41. Also, kappa statistic of more than 0.60 shows a substantial strength of agreement between the actual and predicted classification [83].

**Table 3:** Random forest evaluation metrics.

Metrics	Network types			
	Shelby County	Barabási-Albert	Erdös-Renyi	Newman-Watts-Strogatz
Accuracy	0.82	0.83	0.83	0.83
No Information rate	0.51	0.51	0.52	0.51
Log-loss	0.40	0.38	0.39	0.41
Kappa statistic	0.65	0.66	0.66	0.66
Sensitivity	0.80	0.78	0.77	0.80
Specificity	0.85	0.88	0.89	0.86
Precision (Class 0)	0.84	0.86	0.86	0.84
Precision (Class 1)	0.81	0.81	0.81	0.82
F-Score (Class 0)	0.82	0.82	0.81	0.82
F-Score (Class 1)	0.83	0.84	0.84	0.84

**Fig 3:**
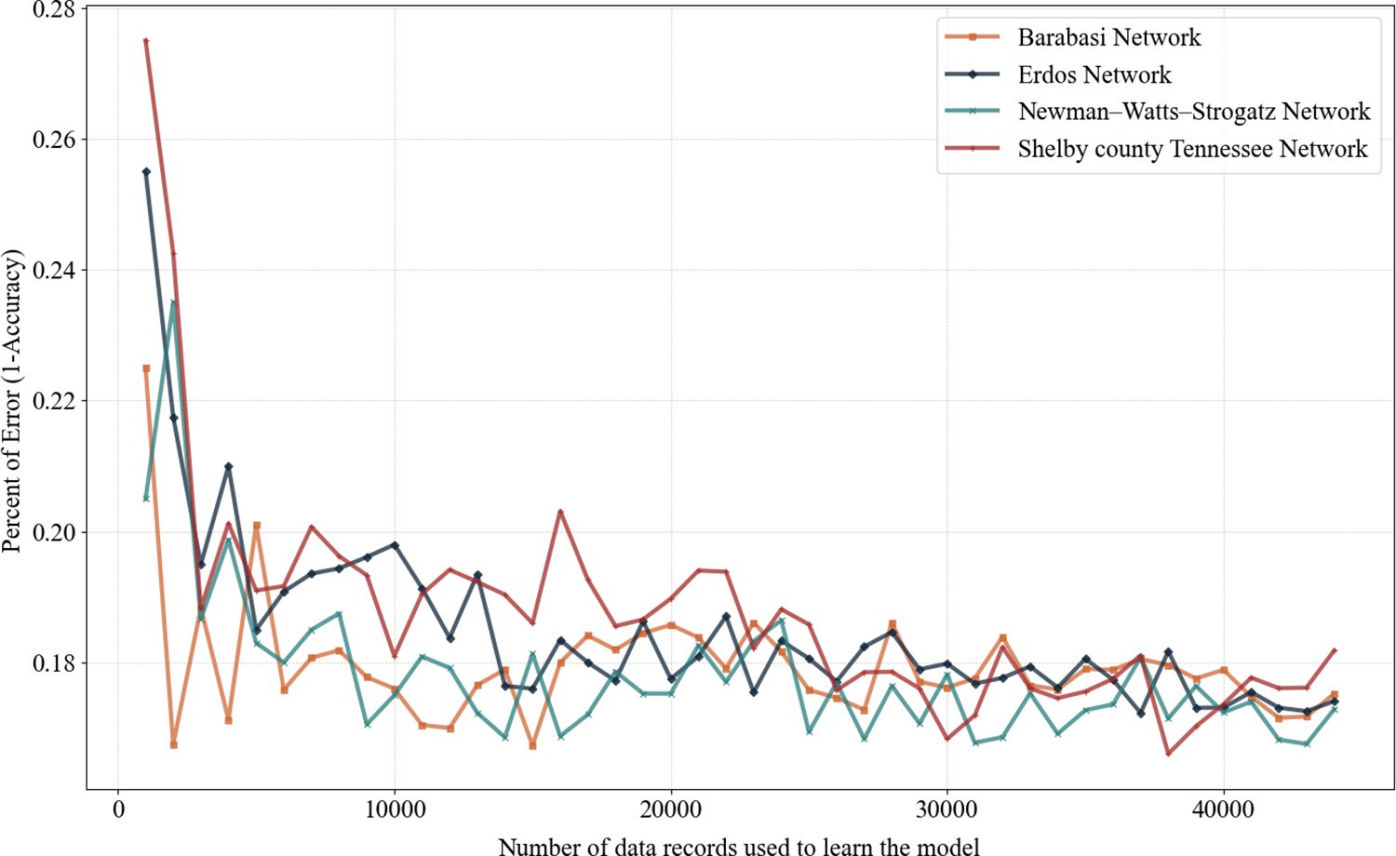
Random forest model accuracy elbow graph.

The number of data required to achieve this level of accuracy has been determined using the elbow graph presented in Fig 3, where the classification model accuracy corresponds to the size of data used for learning purposes. Thus, we divided the data into batches of 1000 instances each and consider the learning data population to increase by a batch for every time that we learn the random forest model. The accuracy of the model for every learned model were recorded and plotted in Fig 3. Although a sharp error reduction can be observed with fewer than 10000 data records, the model’s lack of accuracy appears at its minimum and approaching stability at more than 30000 data records.

Furthermore, the behavior and the performance of the INAR algorithm relative to the structural changes in the network is required to be investigated. The main structural variables that have been considered in this part of the study are: (i) the network size, (ii) the number of the interdependency relationships, and (iii) the network type. Table 4 illustrates an experimental design configuration to explore the effects of these three variables. Note that the Shelby County network was not considered because its characteristics are fixed.

**Table 4:** Experiment design configuration.

Factor	Levels
Number of nodes	2: 60/120
Number of interdependencies	2: 15/30
Network type	3: Barabási-Albert/Erdös-Renyi/Newman-Watts-Strogatz

Performing the full-factorial experiment, according to Table 4, the number of combinations is found by multiplying the numbers of levels for each factor, 2×2×3 = 12. Considering one center point for each network type, the number of combinations increases to 12+3 = 15. As we replicated each treatment twice, the number of observations analyzed is 30. As such, 30 interdependent networks representing various structures have been generated, and data were acquired according to data processing algorithm (Algorithm 1). With these experimental data, the random forest model was learned, and the model’s accuracy was calculated for each repetition. After an initial full ANOVA, only the main effects were significant. The ANOVA was performed again only with the main effects, with results in Table 5.

**Table 5:** ANOVA table for random forest model accuracy.

Source	Sum of squares	df	Mean square	*F* stat	*p*-value
**Model**	**25.25**	**4**	**6.31**	**13.51**	**< 0.0001**
Number of components	3.45	1	3.45	7.38	0.0118
Number of interdependencies	4.35	1	4.35	9.31	0.0053
Network type	17.45	2	8.73	18.67	< 0.0001
**Residual**	**11.69**	**25**	**0.4675**		
Lack of fit	4.19	10	0.4192	0.8389	0.6015
Pure error	7.50	15	0.4997		

**Fig 4:**
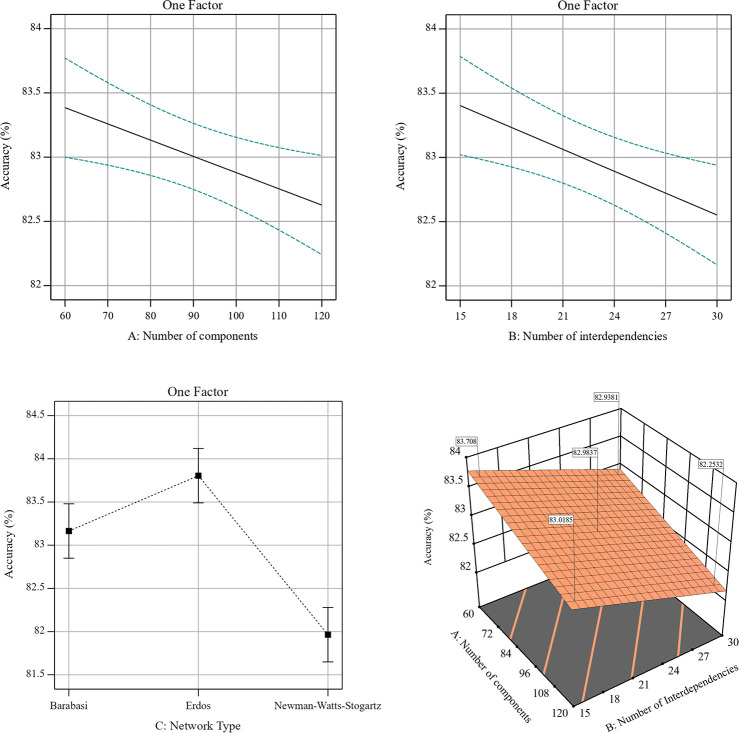
Accuracy versus the (a) number of components, (b) number of interdependencies, (c) network type, as well as (d) the effect of number of components and number of interdependencies on accuracy considering the average over factor C.

The *R*^2^ is 0.5448 (adjusted *R*^2^ of 0.6330) suggests a relationship between the controllable factors and the accuracy of the random forest model, although it explains only the 63% of the response total variance. This experiment’s signal-to-noise ratio, comparing the range of the predicted values to the average prediction error, is 12.359, which is greater than 4, the rule of thumb that suggests an adequate signal [84]. Therefore, the experiment analysis confirms the significance of the main factors and the existence of a linear relationship between factors and the response. Fig 4 illustrates the effects of variation in the three factors. Although the accuracy of the model decreases with an increasing number of components and interdependencies in the network, the degradation of accuracy considering the experiment domain is around just 1%. This suggests that the variation in accuracy does not significantly affect the performance of the entire algorithm.

### Recovery time chain prediction

**Fig 5:**
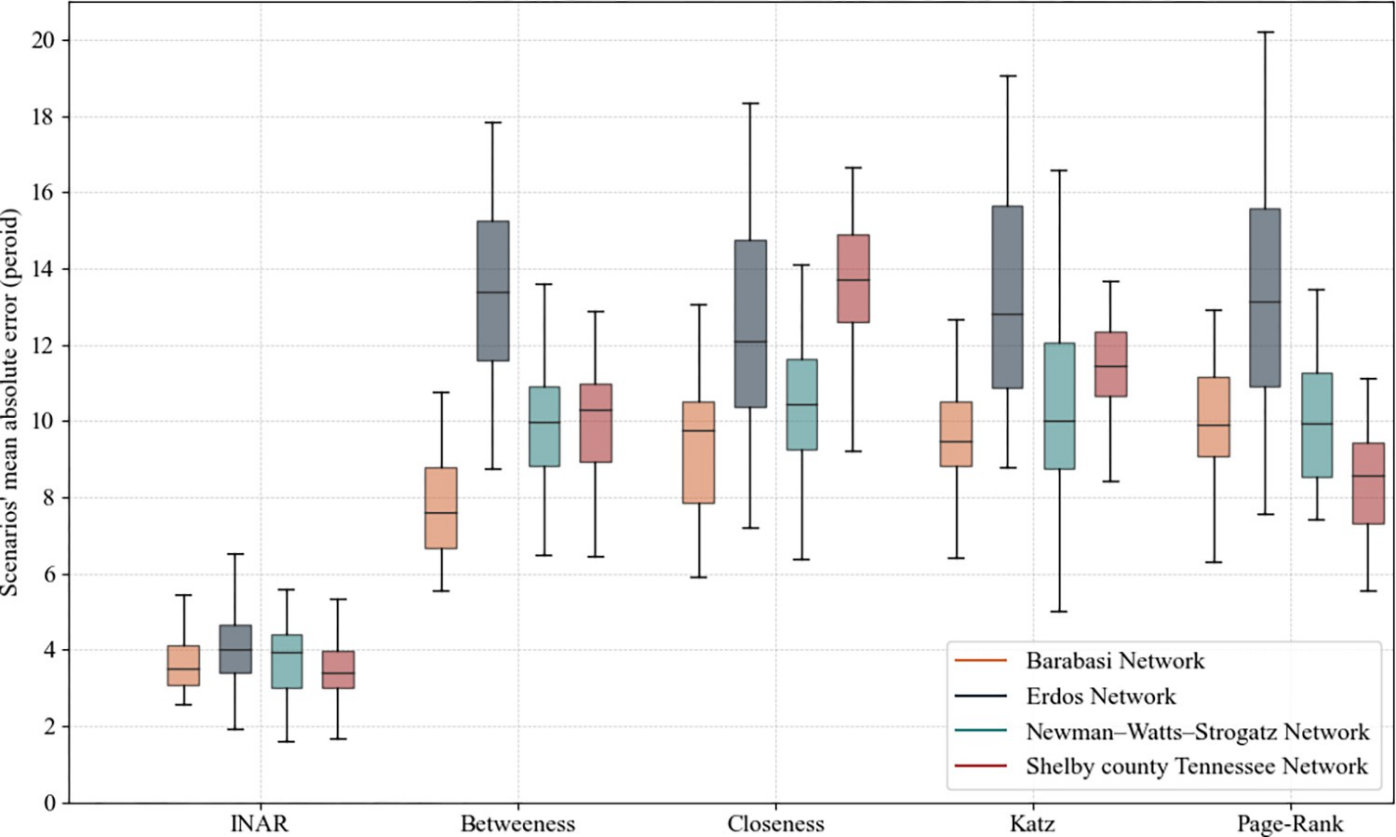
Mean absolute error for the disrupted interdependent component recovery period prediction.

Mentioned previously, in each period, we use the random forest model to predict if each disrupted component is recovered or not in that period. Because the random forest model is not 100% accurate and each node is classified independent from the other components, more than one component can be predicted as recovered in each period. To rectify this problem, we rank the selected components based on the probability generated by the random forest model and select the one component with the highest probability as the component predicted to be restored in a specific period. That is, a short-list of the disrupted components is provided using the random forest model, and the one with the highest score among them is selected. So, we expect better accuracy in predicting the whole chain of disrupted interdependent components than the random forest model’s independent predictions. The boxplots in Fig 5 show the accuracy of the prediction made by the INAR algorithm versus those made using rankings from individual centrality measures. To analyze the performance of the INAR algorithm, it is currently unrealistic to consider a massive number of disruption scenarios and to enumerate all possible combinations due to the computational effort required, mainly when dealing with the large number of disrupted components in an interdependent network. Therefore, we generated 30 random disruption scenarios, along with 40 disrupted components, including 15 and 10 interdependent components for the proposer’s and responder’s networks. Subsequently, we measured how different the predicted restoration and the actual restoration were in terms of the number of periods and lead the discussion using the mean and standard deviation of the random sample. The distributions of mean absolute error (found from the predictions of 15 disrupted components) are found in Fig 5. According to Fig 5, the INAR algorithm has the lowest mean absolute error of 3.49 (standard deviation of 0.9) for the Shelby County network. Similar results were found for the other network types. The performance of the INAR algorithm is substantially better than restoration decisions based on centrality measures across the network types. The use of centrality measures was substantially worse (and more variable) for Erdös-Renyi, which are networks whose connections are generated randomly. Centrality measures worked a bit better for Barabási-Albert networks, whose degree distribution follows a power law, suggesting only a small portion of the components has a high degree distribution. Thus, using centrality measures for decision-making naturally produces better performance.

**Fig 6:**
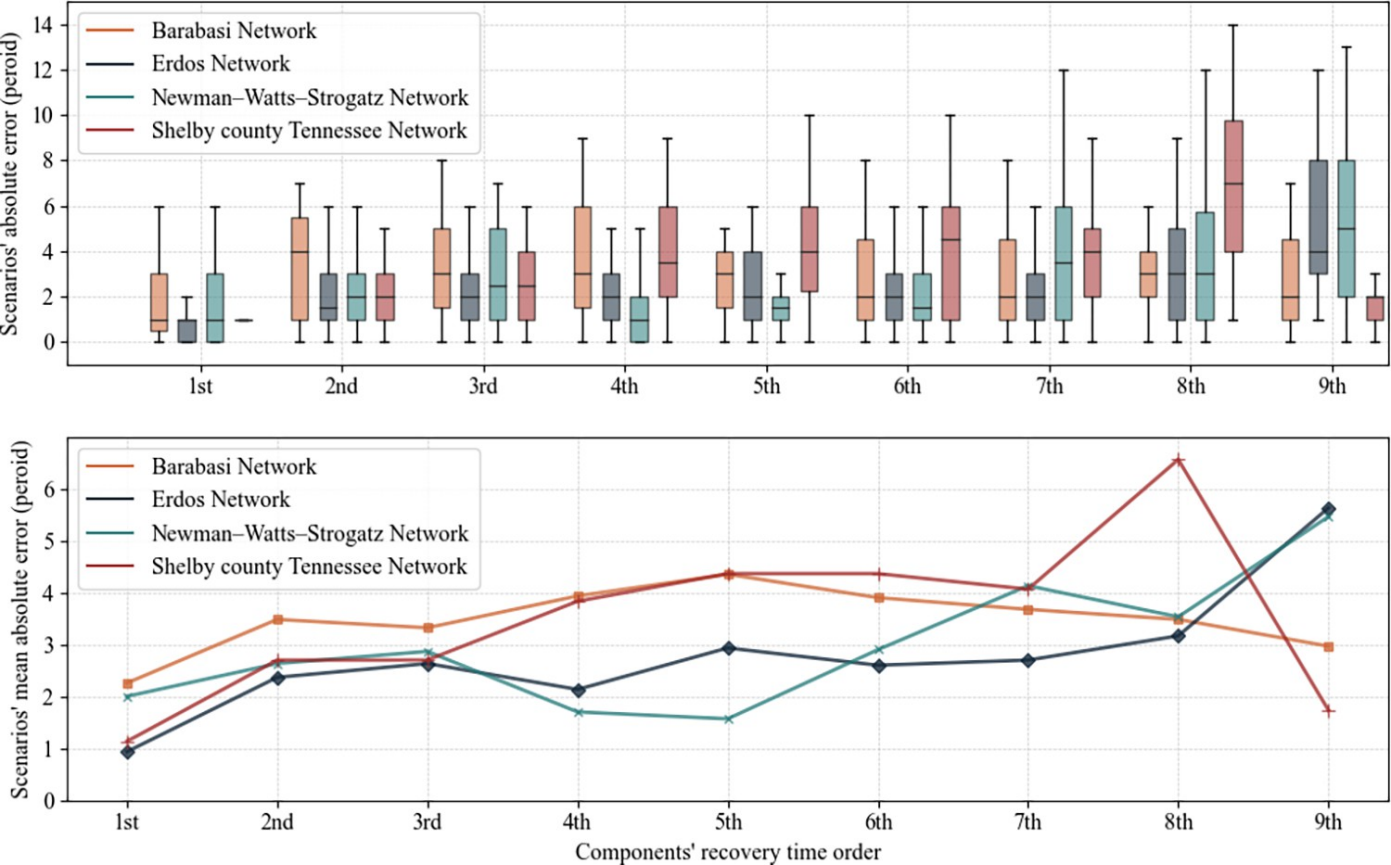
Prediction chain performance based on recovery time order.

Generally, it is easier to predict an incident happening in the near future relative to the distant future, which may be a function of a chain of uncertain events and decisions. That is, if we fail to predict the events happen in the near future correctly, the dependent incidents in the distant future are less likely to be predicted correctly. The INAR algorithm reflects this concept as we are dealing with a finite number of disrupted components that need to be recovered in time. As the INAR algorithm makes predictions period by period, the prediction made in later periods depends on the ones in earlier periods. Thus, as the time passes, we expect to lose accuracy in predicting restoration time. To address this concern, we consider a continuous reproduction process for if the responder realizes a deviation between prediction and the actual plan of the proposer. Therefore, the responder tries to keep the gap between their assumption and what actually happens as close as possible, so predictions made in the future are less important because they will likely be corrected in the algorithm process. In Fig 6, we considered the ascending order of restoration time for each disruption scenario and calculate the absolute error of actual and predicted time of recovery. Doing so shows the model prediction performance corresponding to the proposer’s consecutive order of recovery.

It is evident in Fig 6 that the mean and standard deviation of prediction error generally increases as the component’s recovery order increases. This indicates that the further in time an event happens, the less accuracy can be expected from the prediction model. Also, we can realize that the INAR algorithm has a better performance in predicting the near future with the mean absolute error of 2.25, 0.93, 2, and 1.13 for Barabási-Albert, Erdös-Renyi, Newman-Watts-Strogatz, and Shelby County network, respectively. Using a continuous reprediction mechanism whenever we find deviation between the actual and predicted plan, the INAR algorithm frequently sheds light on the near future and always puts the algorithm in position to predict the prior component that is recovered by the proposer.

### Recovery cost

**Fig 7:**
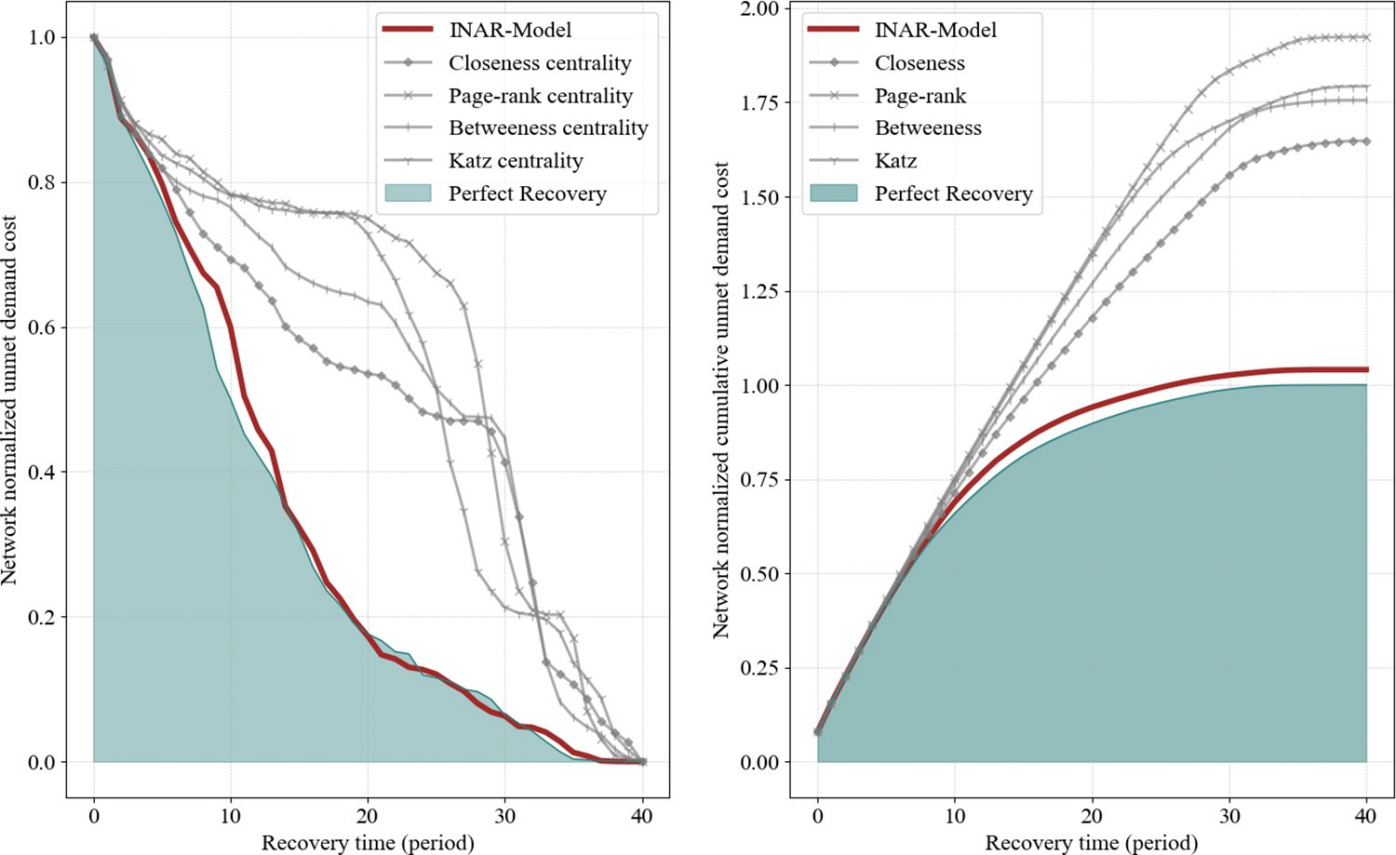
Network normalized unmet demand cost during the recovery process.

We have illustrated the performance and behavior of the prediction of the INAR algorithm in detail. In this section, we address how a lack of accuracy can affect the recovery cost of the responder’s network. We considered the cost of the perfect condition, when the responder knows exactly how the opponent would behave, as the baseline for cost comparisons. Then we calculate the network unmet demand cost considering (i) the prediction made using INAR algorithm and (ii) by ranking disrupted component recovery time based on individual centrality metrics. To have a realistic point of view, Fig 7 shows the average unmet demand cost considering 20 different disruption scenarios during the network recovery time horizon for the Shelby County network.

Illustrated in Fig 7, the recovery cost related to the INAR algorithm is closely aligned with the perfect recovery condition, suggesting that the network recovery solution provided by the INAR algorithm can successfully compete with the optimal solution. Furthermore, the same trend can be seen for other generated networks that represent different network structures in this study. Considering the perfect situation as the baseline, we calculate the percentage of the extra cost imposed due to the lack of accuracy in predictions made by utilizing the above-mentioned methods for various type of networks. Table 6 illustrates the optimal cumulative recovery cost as the baseline, and the amount of extra cost that imposed due to using different prediction algorithms. The cost deviation from the baseline optimal cumulative cost considering the INAR algorithm for generated networks is between 1% to 3%, while this amount for the Shelby County network is almost 4%. Evident from the Table 6, the INAR algorithm shows a better cost-performance than the other ranking methods considering the realistic Shelby County network. Therefore, Table 6 confirm the high performance of the INAR algorithm compared to the other prediction methods, especially for the Shelby County network, which is based on a real-world topology of actual interdependent infrastructure networks.

**Table 6:** Cumulative recovery cost of recovery.

			Percent of extra cost due to the lack of accuracy			
Network type	Baseline optimal cumulative cost	INAR algorithm	Betweenness centrality ranking	Closeness centrality ranking	Page-rank centrality ranking	Katz centrality ranking
Shelby County	456,449,810	3.98%	48.24%	39.11%	59.29%	52.60%
Barabási-Albert	349,182,870	1.27%	3.52%	4.21%	5.13%	4.56%
Erdös-Renyi	426,206,130	2.76%	7.80%	8.92%	6.98%	8.47%
Newman-Watts-Strogatz	80,207,450	1.09%	7.11%	7.42%	6.98%	6.86%

Note from Table 7 that the running time of Algorithm 2 for the prediction and optimization process was insignificant relative to the time scale of the decision process and also similar across the selected network types.

**Table 7:** Computation time (seconds) for Algorithm 2 by network type.

Network type	Shelby County	Barabási-Albert	Erdös-Renyi	Newman-Watts-Strogatz
Computational time	52	54	47	39

## Concluding remarks

The INAR algorithm is developed to address the concern of having multiple infrastructure decision-makers whose decisions affect (i) the performance of other infrastructures and thus (ii) the decisions of other infrastructure decision-makers. This is especially true when these decision-makers do not reveal their restoration plans to other players in the system. The INAR algorithm provides a framework for predicting future decision-maker behavior and devising an adaptive plan concerning the recovery of the disrupted interdependent infrastructure network. To do so, we have integrated machine learning with an analytical solution approach by defining a mechanism to include uncertain assumption (i.e., the best guesses of the intentions of other players) into the analytical model.

The main idea behind the INAR algorithm development objective is to use topological feature of the proposer’s network, investigating and learning from disruption scenarios before they occur, so that a good solution can be achieved in a timely manner after a disruption. The proposed algorithm consists of two parts: (i) data preparation and learning using a random forest model and (ii) scheduling the recovery process. The prediction model showed good performance when predicting the recovery periods of each individual component in the proposed case studies, which include the system of interdependent water and power networks from Shelby County, TN, and three systems with different types of randomly generated networks: Barabási-Albert, Erdös-Renyi, and Newman-Watts-Strogatz.

Furthermore, we have studied the recovery prediction accuracy of the disrupted interdependent components complete sequence using the mean absolute error between actual and predicted plan. The results showed a high performance with an average deviation of around 4 periods from actual, while this amount for the near feature was around 2 periods. In this regard, we observed myopic behavior from the INAR algorithm prediction, which has been addressed using a continuous prediction loop to renew the predictions whenever a deviation was detected by the algorithm. Finally, we addressed the reflection of the prediction lack of accuracy objectively on the cost of the recovery. Using the INAR algorithm, unmet demand recovery costs approach the optimal solution recovery cost such that we can observe only a 4% cost deviation from the optimal solution for Shelby County case and less than 3% for the other generated networks. This implies a high efficiency of the proposed algorithm. The challenge for the future work is to enhance the overall performance of the algorithm, particularly when predicting the distant future events in the proposer’s network. Deep learning models can help achieve better predictions and improving the algorithm overall performance. Furthermore, the proposed algorithm is suitable for the real cases like hierarchical systems where the responder player does not have that much leverage over the proposer player in the system. Incorporating different levels of bargaining power for each player in the system could be other relevant future avenue of this research.
